# Multi‐trait genomic selection improves the prediction accuracy of end‐use quality traits in hard winter wheat

**DOI:** 10.1002/tpg2.20331

**Published:** 2023-05-17

**Authors:** Harsimardeep S. Gill, Navreet Brar, Jyotirmoy Halder, Cody Hall, Bradford W. Seabourn, Yuanhong R. Chen, Paul St. Amand, Amy Bernardo, Guihua Bai, Karl Glover, Brent Turnipseed, Sunish K. Sehgal

**Affiliations:** ^1^ Department of Agronomy, Horticulture and Plant Science South Dakota State University Brookings South Dakota USA; ^2^ USDA‐ARS, CGAHR Hard Winter Wheat Quality Laboratory Manhattan Kansas USA; ^3^ USDA‐ARS Hard Winter Wheat Genetics Research Unit Manhattan Kansas USA

## Abstract

Improvement of end‐use quality remains one of the most important goals in hard winter wheat (HWW) breeding. Nevertheless, the evaluation of end‐use quality traits is confined to later development generations owing to resource‐intensive phenotyping. Genomic selection (GS) has shown promise in facilitating selection for end‐use quality; however, lower prediction accuracy (PA) for complex traits remains a challenge in GS implementation. Multi‐trait genomic prediction (MTGP) models can improve PA for complex traits by incorporating information on correlated secondary traits, but these models remain to be optimized in HWW. A set of advanced breeding lines from 2015 to 2021 were genotyped with 8725 single‐nucleotide polymorphisms and was used to evaluate MTGP to predict various end‐use quality traits that are otherwise difficult to phenotype in earlier generations. The MTGP model outperformed the ST model with up to a twofold increase in PA. For instance, PA was improved from 0.38 to 0.75 for bake absorption and from 0.32 to 0.52 for loaf volume. Further, we compared MTGP models by including different combinations of easy‐to‐score traits as covariates to predict end‐use quality traits. Incorporation of simple traits, such as flour protein (FLRPRO) and sedimentation weight value (FLRSDS), substantially improved the PA of MT models. Thus, the rapid low‐cost measurement of traits like FLRPRO and FLRSDS can facilitate the use of GP to predict mixograph and baking traits in earlier generations and provide breeders an opportunity for selection on end‐use quality traits by culling inferior lines to increase selection accuracy and genetic gains.

## Core Ideas

- Improvement of end‐use quality is one of the most important goals in hard winter wheat breeding.
- Multi‐trait genomic prediction (MTGP) can be used to predict end‐use quality traits that are difficult to phenotype.
- Including rapid and NIR traits into MTGP improves prediction accuracy for end‐use quality traits.
- MTGP can provide an opportunity for selection on end‐use quality traits in earlier generations.

**Abbreviations**
AACC: American Association of Cereal Chemists International
BLUE: best linear unbiased estimate
GBLUP: genomic best linear unbiased predictor
GBS: genotyping‐by‐sequencing
GP: genomic prediction
GS: genomic selection
HWW: hard red winter wheat
MT: multi‐trait
MTGP: multi‐trait genomic prediction
MTGS: multi‐trait genomic selection
NIR: near‐infrared
NIRS: near‐infrared spectroscopy
PA: prediction accuracy
SNP: single‐nucleotide polymorphism
STGP: single‐trait genomic prediction

## 1 INTRODUCTION

Hard winter wheat (HWW; *Triticum aestivum* L.) is the major wheat class grown in the United States and accounts for about 46% of the total wheat production in the country (USDA NASS, 2021). This versatile class of wheat exhibits excellent milling and baking characteristics suitable for a variety of wheat foods, especially bread. Owing to high demand, most of the US‐produced HWW is exported. For instance, 52% of the total HWW produced in 2020 was exported worldwide (USDA ERS, 2022). Throughout the wheat supply chain, end‐use quality characteristics play an important role in the marketing and pricing of HWW (Roberts et al., 2022). Moreover, consumers’ preferences for healthier food necessitate an emphasis on the selection for desirable end‐use quality traits. Thus, wheat breeders must improve end‐use quality traits while simultaneously breeding for increased yield to meet projected demand.

The high gluten strength and damaged starch in HWW make it very suitable for baking, and yeast‐leavened bread is a major end‐use product. Bread quality is an important but complex trait that is defined by a combination of many parameters (Battenfield et al., 2016). Several important factors, including kernel characteristics, the milled flour quality, protein and starch strength, and dough properties, all play a crucial role in determining the end‐use quality of the final product. Hence, several assays are used to profile these factors and inform the selection for end‐use quality. Nevertheless, most of the assays for the evaluation of end‐use products are expensive, time‐consuming, and require large quantities of flour. Therefore, breeders mostly prioritize the selection for agronomic traits and disease resistance in earlier generations and for quality traits in advanced generations in most breeding programs (Battenfield et al., 2016). Previous studies have shown that end‐use quality traits are controlled by a few major genes and a large number of quantitative trait loci with minor effects (Carter et al., 2012; Jernigan et al., 2018; Kiszonas & Morris, 2017; Sandhu, Aoun, et al., 2021 ; Sandhu, Patil, et al., 2021 ). Though available major genes have been exploited in breeding programs, the majority of minor genes are highly influenced by the environment and remain uncharacterized (Jernigan et al., 2018; Kiszonas & Morris, 2017). Thus, genomic selection (GS) is a potentially effective tool to assist in the selection for end‐use quality traits in earlier generations.

GS employs whole‐genome marker information to predict the breeding value of an individual (Heffner et al., 2009; Meuwissen et al., 2001). Further, GS has been shown to increase genetic gain per breeding cycle and improve selection accuracy for complex traits (Bassi et al., 2015; Juliana et al., 2019), particularly the traits that are expensive and difficult to phenotype and cannot be evaluated at earlier stages of the breeding program (Battenfield et al., 2016; Gill et al., 2021). In recent years, GS has been evaluated for the prediction of various complex traits in wheat, including agronomic traits (Gill et al., 2021; Juliana et al., 2020; Rutkoski et al., 2016; Ward et al., 2019), disease resistance (Juliana et al., 2017; Rutkoski et al., 2012; Zhang et al., 2022), and end‐use quality (Battenfield et al., 2016; Ibba et al., 2020; Lado et al., 2018; Sandhu, Aoun, et al., 2021; Sandhu, Patil, et al., 2021; Zhang‐Biehn et al., 2021). Most of these studies showed success in GS and suggested the possibility for use of GS to predict complex traits in wheat breeding. Although numerous studies have evaluated GS for end‐use quality in wheat, most employed soft wheat germplasm with only one study using HWW from the US Great Plains (Zhang‐Biehn et al., 2021). As the processing methods and end‐use objectives are quite different among different classes of wheat, it is necessary to further evaluate the usability of GS to predict various milling and baking traits in HWW.

**Core Ideas**

- Improvement of end‐use quality is one of the most important goals in hard winter wheat breeding.
- Multi‐trait genomic prediction (MTGP) can be used to predict end‐use quality traits that are difficult to phenotype.
- Including rapid and NIR traits into MTGP improves prediction accuracy for end‐use quality traits.
- MTGP can provide an opportunity for selection on end‐use quality traits in earlier generations.

Most of the GS studies primarily used single‐trait genomic prediction (STGP) models for predicting individual traits. In recent years, several simulated and empirical studies evaluated multi‐trait (MT) genomic prediction (GP) models that can leverage genetic correlations among different traits to improve prediction accuracy (PA) for traits(s) of interest (Gaire et al., 2022; Gill et al., 2021; Jia & Jannink, 2012; Lado et al., 2018; Zhang et al., 2022). In most of these studies, multi‐trait genomic prediction (MTGP) models showed superior performance to the conventional STGP models. Few studies evaluated the MT models to evaluate GS for end‐use quality traits in wheat (Hayes et al., 2017; Lado et al., 2018; Michel et al., 2018; Sandhu et al., 2022; Zhang‐Biehn et al., 2021). However, only Zhang‐Biehn et al. (2021) evaluated MTGP for HWW end‐use quality traits and used few prebaking assays as covariates to predict bread quality. Henceforth, the further evaluation of MTGP to predict end‐use quality traits in HWW may facilitate its implementation in HWW breeding programs. In addition, several grain quality assays such as hybrid sodium dodecyl sulfate–solvent retention capacity (SDS–SRC) and flour characteristics that require minimal resources and can be evaluated in early breeding generations show a high association with primary end‐use quality traits (Seabourn et al., 2012). Evaluating the MTGP models that incorporate those traits to predict baking traits may help to predict end‐use traits in the early stages of breeding programs.

**FIGURE 1:**
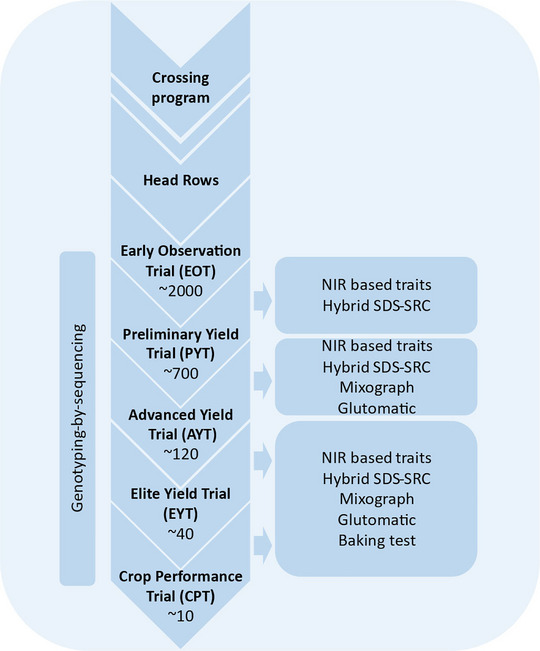
Schematic representation of the South Dakota State University winter wheat breeding program. The early stage of yield trials is the Preliminary Yield Trial (PYT, ∼700 lines) advanced from Early Observation Trial (EOT) consisting of short rows derived from selected single plants. The PYT is followed by Advanced Yield Trial (AYT), Elite Yield Trial (EYT), and state‐wide Crop Performance Testing (CPT) nursery. The quality assessment starts from the PYTs, and various quality assays are used at different stages of development owing to the availability of flour and other resources. The different quality assays performed at various stages of the breeding program are elucidated in the figure.

Depending on the availability of flour and other resources, different types of quality tests are used to assess the end‐use traits and inform selections in different trials in breeding programs, including South Dakota State University winter wheat breeding program (Figure 1). In early generations, the grain and flour characteristics are assessed using a near‐infrared (NIR) reflectance–based analyzer followed by a rapid and small‐scale hybrid SDS–SRC test to predict breadmaking quality. Additional tests like mixograph analysis for rheological properties and Glutomatic analysis for gluten quantity and quality determination are also included in advanced generations, and lastly, complete end‐use profiling, including baking tests, is conducted in the final stages of the breeding programs (Figure 1). As it is challenging to perform a mixograph or actual baking tests in earlier stages, such as Preliminary Yield Trials (PYT), MTGP could be employed to predict baking traits at these stages. The MTGP models can be informed with trait measurements from rapid assays in PYTs along with complete data from more advanced trials to predict end‐use quality traits with higher selection accuracy. This necessitates the evaluation of different trait combinations from different assays that can help in improving the PA of baking traits using MTGP. In this study, we used a set of breeding lines from the advanced breeding trials of the SDSU winter wheat program that were evaluated for a variety of quality parameters, including milling, processing, and baking characteristics, and genotyped using the genotyping‐by‐sequencing (GBS) approach. The objectives of this study were to (i) evaluate GS using single‐trait and MT models for different end‐use traits and (ii) explore the usability of rapid, small‐scale, and NIR spectroscopy (NIRS)‐based traits as covariates to inform MTGP models for baking quality.

## 2 MATERIALS AND METHODS

### 2.1 Plant materials and phenotyping

In this study, we used a set of lines evaluated in SDSU Elite Yield Trials (EYT) and Crop Performance Trials (CPT) nurseries from 2015 to 2021 that were profiled for a variety of end‐use quality traits, including baking tests. During this period, a total of 300 samples, including checks, were evaluated in these nurseries. The EYT and CPT nurseries were planted at multiple locations each year in a randomized complete block design with three and four replications, respectively, along with a set of check cultivars (“Alice,” “Expedition,” “Lyman,” “Overland,” “Redfield,” and “Winner”). Each year, lines from two locations were used for end‐use quality profiling, including baking. In addition to checks, a set of about 15 lines was shared among every 2 years as these lines were advanced from EYT to CPT nurseries. For instance, 18 lines were common between 2015 and 2016, whereas 19 lines overlapped between 2016 and 2017. Overall, 176 unique lines were evaluated for milling and baking tests from 13 environments (site‐year combination) from 2015 to 2021. A detailed description of the lines used in this study is provided in Table S1. Since the short harvest‐to‐planting interval of less than 1 month in SD, the quality traits of the lines are analyzed using grain harvested from preceding nursery seasons.

A total of 14 processing and end‐use quality traits were assessed using various assays. Owing to limited test capacity, grain from replications within locations was pooled into a single sample for milling and baking test profiles. Grain protein content (GRPROT) was determined using NIR analysis following AACC‐approved methods 39‐10.01 (AACC International, 2011) and adjusted to a 13% moisture equivalent. Grain samples were tempered to the required moisture (14%) and milled using a Brabender Quadrumat Senior laboratory mill at the USDA Hard Winter Wheat Quality Laboratory (HWWQL), in Manhattan, KS. The milled product was weighed to determine fraction yield on a total product, thus providing an estimation of flour yield (FLRYLD). Flour characteristics, including protein (FLRPROT) and ash (FLRASH), were analyzed using NIR following AACC methods 39‐11.01 and 08‐21.01 (AACC International, 2011). These traits were further adjusted to 14% moisture content equivalence. Mixograph analysis was performed using Mixgraph (National Manufacturing Company, USA) based on AACC‐approved method 54‐40.02 (AACC International, 2011) to estimate the water absorption (MIXABS), optimum development time (MIXTIM), and tolerance to overmixing (MIXTOL). Glutomatic analysis was performed using a Perten Glutomatic 2000 system following AACC 38‐12.02 (AACC International, 2011) to estimate wet gluten content (WGC) and gluten index (GI) as described in the AACC method. Further, we used WGC and GI to determine another index for wet gluten (WGI) using the formula (WGC × GI)/100. These analyses were performed using two replicates per sample. SDS sedimentation was performed using the modified hybrid SDS–SRC method (Seabourn et al., 2012) at SDSU Crop Quality Lab using the residual flour samples for all the entries analyzed for baking. Briefly, the hybrid SDS–SRC method combines the SDS sedimentation method (AACC 56‐70) (AACC International, 2000) and SRC method (AACC 56‐11) (AACC International, 2000) to estimate flour sedimentation weight value (FLRSDS) in percent using the formula described in Seabourn et al. (2012). To evaluate the end‐use quality of yeast‐leavened bread, a pan‐bread was baked as pup loaves following the AACC method 10‐10.03 (AACC International, 2011). Optimum water absorption from baking tests was recorded as bake absorption (BAKEABS). Loaf volume (LVOL) in cc and specific loaf volume (SpLVOL) in cc/g were recorded after baking by using the rapeseed displacement method 10‐05.01 (AACC International, 2011). Samples were baked in two replicates, and means were used in the final analysis.

### 2.2 Genotyping

The set of breeding lines used in this study was genotyped using the GBS approach at the USDA Central Small Grain Genotyping Lab, Manhattan, KS, as described in Gill et al. (2022). Briefly, the DNA was isolated from each line using fresh leaf tissue at the three‐leaf stage using a modified cetyltrimethyl ammonium bromide method (Bai et al., 1999). The GBS libraries were prepared using double‐restriction digestion with HF‐*PstI* and *MspI* restriction enzymes (Poland et al., 2012) and sequenced on an Ion Proton sequencer (Thermo Fisher Scientific, Waltham, MA, USA) or NextSeq 500 (Illumina Inc, USA). Single‐nucleotide polymorphism (SNP) variants were called using the GBS v2.0 SNP discovery pipeline in TASSEL v5.0 (Bradbury et al., 2007) using the Chinese Spring wheat genome reference RefSeq v2.0 (IWGSC, 2018; Zhu et al., 2021). For quality control, SNPs were filtered to remove the markers with >30% missing calls, <5% minor allele frequency, and >10% heterozygosity. The remaining SNPs were imputed using BEAGLE v4.1 (beagle.27Jan18.7e1.jar; https://faculty.washington.edu/browning/beagle/b4_1.html) (Browning & Browning, 2007) for further analyses.

### 2.3 Statistical analysis

The best linear unbiased estimates (BLUEs) of each line for the majority of the traits were estimated as described by Zhang‐Biehn et al. (2021) using the following model:

$$Y_{\mathrm{ij}}\;=\;\mu \;+\;\mathrm{Lin}e_{i}+\;\mathrm{En}v_{j}+\;e_{\mathrm{ij}}$$

where *Y_ij_* is the phenotypic value of the *i*th line in the *j*th environment (site‐year combination), *μ* is the overall mean, *Line_i_* is the fixed effect of the *i*th line, *Env_j_* was the random effect of the *j*th environment, and *e_ij_* is the residual error for genotype the *i*th line in *j*th environment. The BLUEs for Glutomatic traits were estimated using the following model:

$$Y_{\mathrm{ijk}}\;=\;\mu \;+\;\mathrm{Lin}e_{i}+\;\mathrm{En}v_{j}+\;\mathrm{Rep}{\left(\mathrm{Env}\right)}_{\mathrm{jk}}\;+\;e_{\mathrm{ijk}}$$

where *Y_ijk_* is the observed phenotypic, *μ* is the overall mean, *Line_i_* is the fixed effect of *i*th line, *Env_j_* was the random effect of *j*th environment, and *Rep(Env)_k_* is the random effect of *k*th replicate within the *j*th environment, and *e_ij_* is the residual error term.

To extract variance components, best linear unbiased prediction (BLUP), and estimate broad‐sense heritability, line effects were fitted as random in the above equations. Broad‐sense heritability was estimated with Cullis's method (Cullis et al., 2006) using the following equation:

$$\;H_{Cullis}^{2}=\;1-\frac{{\bar{v}}_{\Delta ..}^{BLUP}}{2\sigma_{g}^{2}}$$

where ${\bar{v}}_{\Delta ..}^{BLUP}$ is the mean‐variance of the difference of two BLUPs for the line effect, and $\sigma_{g}^{2}$ is the genotypic variance. The above‐described equations were implemented using ASReml‐R Version 4.0 (Butler et al., 2018) in the R programming language (R Core Team, 2018). The summary statistics, pairwise comparisons, and principal component analysis (PCA) were performed in R using custom scripts or different packages, including psych and ggplot2 (Wickham, 2016; William, 2013).

### 2.4 Genomic prediction models

#### 2.4.1 Single‐trait GP models

Three different ST models were compared to select the best performing model as a baseline for comparison with MTGP model. The first ST model was standard genomic best linear unbiased prediction (GBLUP) employing a genomic relationship (*G*) matrix implemented using the following equation:

y=μ+Zg+e

where *y* is the vector (*n* × 1) of BLUE values for each trait; *μ* is the overall mean, *Z* is the incidence matrix for genotype effects; *g* is a vector of normally distributed marker predictor effects with $g\;∼\;N(0,\;G\sigma_{g}^{2})$, where *G* is the genomic relationship matrix (VanRaden, 2008) $\sigma_{g}^{2}$ is the additive genetic variance; and *e* is the vector of residual errors with $e\;∼\;N(0,\;\sigma_{e}^{2})$.

In addition to GBLUP, we used two Bayesian models, Bayes A (BA) and Bayes B (BB), which assume different prior distributions for estimating marker effects and variances (Pérez & De Los Campos, 2014). The Bayes A model uses the scaled inverse chi‐squared probability distribution for estimating marker variances. Bayes B is an extension of the Bayes A model (Meuwissen et al., 2001) and employs an inverse chi‐square distribution for marker effects and assumes that some markers have no effect. The Bayesian models were implemented as follows:

$$\;y_{i}=\mu +\sum_{j\;=\;1}^{j\;=\;p}x_{ij}\beta_{j}+e_{i}$$

where *y* refers to BLUE values for each trait; *μ* is the overall mean; $x_{ij\;}$is the identity of the SNP marker, $\beta_{j}\;$is the marker effect, and $e_{i}$ represents the residual error term. The ST models were implemented with 5000 burn‐ins and 25,000 iterations of the Gibbs sampler in R package BGLR (https://github.com/gdlc/BGLR‐R/blob/master/inst/md/GBLUP.md; Pérez &e Los Campos, 2014).

#### 2.4.2 Multi‐trait GP models

We used a Bayesian Multivariate Gaussian model to implement multivariate GBLUP for various traits. The MT model can be expressed using the following equation:

$$\;\left[\begin{array}{c} y_{1}\\ \vdots \\ y_{n} \end{array}\right]=\;\left[\begin{array}{c} I\\ \vdots \\ 0 \end{array}\;\begin{array}{c} 0\\ \vdots \\ I_{n} \end{array}\right]\;\left[\begin{array}{c} \mu_{1}\\ \vdots \\ \mu_{n} \end{array}\right]+\left[\begin{array}{c} Z\\ \vdots \\ 0 \end{array}\;\begin{array}{c} 0\\ \vdots \\ Z_{n} \end{array}\right]\;\left[\begin{array}{c} g_{1}\\ \vdots \\ g_{n} \end{array}\right]+\left[\begin{array}{c} e_{1}\\ \vdots \\ e_{n} \end{array}\right].$$

where *y* is the *n*‐dimensional vector of BLUEs for n traits, *I* and *Z* are the design matrices, $\mu_{t},$ *t* = 1⋯*n*, refers to trait intercepts of n traits, $\left[\begin{array}{c} g_{1}\\ \vdots \\ g_{n} \end{array}\right]$ are the predicted genetic values assumed to be distributed as ∼MVN(0,∑⊗G) with *G* representing the genomic relationship matrix obtained following VanRaden (2008), and ⊗ refers to the Kronecker product of two matrices. The residuals of the MT model were assumed to be distributed as $\left[\begin{array}{c} e_{1}\\ \vdots \\ e_{n} \end{array}\right]$ ∼MVN(0,R⊗I). The matrices ∑ and *R* are the variance–covariance matrices for the genetic and residual effects between traits, respectively, where ∑ was estimated as an unstructured variance–covariance matrix and R as a diagonal variance–covariance matrix. The multitrait GBLUP was implemented in the MTM package in R (de los Campos & Grüneberg, 2016) using the Gibbs sample algorithm with 5000 burn‐ins and 25,000 iterations. The abovementioned model was also used to estimate the genetic correlation between different traits.

#### 2.4.3 Combination of traits for multi‐trait GP models

The MTGP model was evaluated for the prediction of mixograph and baking traits using various combinations of secondary traits. For the mixograph predictions, three traits, including MIXABS, MIXTIM, and MIXTOL, were used as primary traits. Similarly, three baking traits, namely, BAKEABS, LVOL, and SpLVOL, were used as primary traits. To predict these primary traits using the MT model, we evaluated the inclusion of different sets of secondary traits from different quality assays (Figure S1). For instance, we compared the incorporation of grain characteristics, flour characteristics, Glutomatic assay, flour SDS, or Mixograph traits as secondary traits to predict LVOL. Likewise, these sets of secondary traits were used to assess the PA of the MT model for different primary traits.

### 2.5 Cross‐validation of the GS models

The PA of the GS models was deduced by calculating the correlation between genome‐estimated breeding values (GEBVs) and the actual phenotypic values of individuals in a validation set through a cross‐validation approach. We used 100 random sets of 5‐fold CV approach to evaluate the PA of ST and MT models using 2 different validation schemes (CV1 and CV2). These validation schemes were designed based on real scenarios observed in plant breeding experiments. The CV1 was used to evaluate the ST models, where four of the fivefolds (80%) were used as the training set (had genotypic and phenotypic data) to train the model, and the remaining fold (20%) was used as the validation set (only genotypic data) for prediction. The MT model was evaluated using the CV2 scheme, in which lines were split into fivefolds of equal sizes, with fourfolds as the training set, and the remaining fold as the validation set. To train the model, we used genotypic data and the phenotypic data of the primary trait for the training set, along with phenotypic data of the secondary traits for training as well as the testing set with the objective of predicting the primary trait of the testing set. Figure S2 illustrates various validation schemes used for both models.

## 3 RESULTS

### 3.1 Phenotypic analyses and trait correlations

**FIGURE 2:**
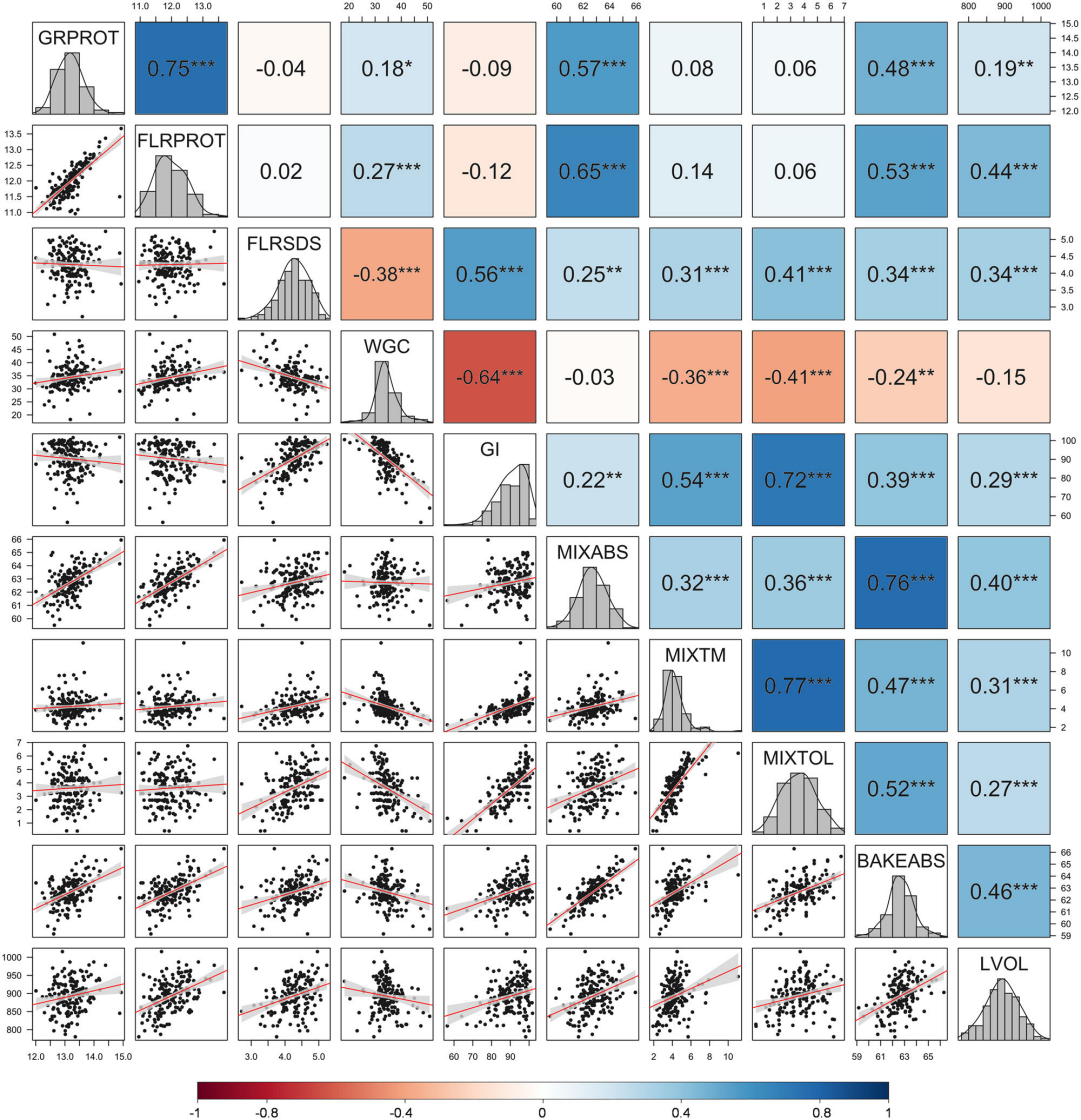
Correlation coefficients among investigated traits using the best linear unbiased estimates (BLUEs) obtained from a multi‐environment analysis. The diagonal of the pair plot elucidates the frequency distribution for various traits, and the lower triangle shows the bivariate scatterplots with fitted lines. Statistically significant differences are denoted by an asterisk (*) where *p ≤ 0.05, **p ≤ 0.01, and ***p ≤ 0.001. BAKEABS, bake absorption; FLRPROT, flour protein content; FLRSDS, flour sedimentation weight; GI, gluten index; GRPROT, grain protein content; LVOL, pup loaf volume; MIXABS, mixing absorption; MIXTM, optimum mix time; MIXTOL, mixograph mix tolerance; WGC, wet gluten content.

The BLUEs for various end‐use quality traits were obtained from multi‐environment analysis to correct for the environmental effects. An approximate normal distribution of obtained BLUEs was observed for most of the traits (Figure 2). Broad‐sense heritability estimates were moderate to high for different traits, ranging from 0.44 to 0.88 (Table 1), with low heritability for GRPROT (0.44) and FLRPROT (0.48) and high heritability for MIXTOL (0.84) and MIXTM (0.88). Significant phenotypic correlations were observed among different quality traits (Figure 2; Table S2). A high positive correlation was observed between GRPROT and FLRPROT (Figure 2). FLRPROT also showed a high positive correlation with MIXABS (0.65; *p* ≤ 0.001), BAKEABS (0.53; *p* ≤ 0.001), and LVOL (0.44; *p* ≤ 0.001). FLRSDS was the only trait that exhibited significant correlations with most of the quality traits, including LVOL (0.34; *p* ≤ 0.001). BAKEABS showed a positive correlation with mixograph traits, including MIXABS (0.76; *p* ≤ 0.001), MIXTM (0.47; *p* ≤ 0.001), and MIXTOL (0.52; *p* ≤ 0.001). Similarly, LVOL was positively correlated with MIXABS (0.40; *p* ≤ 0.001) and MIXTM (0.31; *p* ≤ 0.001).

**TABLE 1:** Descriptive statistics and broad‐sense heritability (*H* ^2^) for different end‐use quality traits.

Trait	Mean	SD	*H* ^2^
GRPROT	13.19	0.47	0.44
FLRYLD	68.90	1.29	0.74
FLRASH	0.42	0.03	0.77
FLRPROT	11.93	0.49	0.48
FLRSDS	325.88	43.89	0.70
MIXABS	62.72	1.06	0.55
MIXTM	4.24	1.15	0.88
MIXTOL	3.61	1.27	0.84
WGC	34.07	4.19	0.71
GI	90.10	7.48	0.84
WGI	30.74	2.80	0.72
BAKEABS	62.66	1.12	0.50
LVOL	892.71	44.21	0.59
SpLVOL	5.95	0.30	0.59

Abbreviations: BAKEABS, bake absorption; FLRASH, flour ash (%); FLRPROT, flour protein content (%); FLRSDS, flour hybrid SDS–SRC sedimentation (weight value %); FLRYLD, flour yield (% recovered); GI, gluten index; GRPROT, grain protein content (%); LVOL, pup loaf volume (cm^3^); MIXABS, mixograph mixing absorption (%); MIXTM, mixograph mix time (min); MIXTOL, mixograph mix tolerance score; SpLVOL, specific loaf volume by weight (g/cm^3^); WGC, wet gluten content; WGI, wet gluten index.

**FIGURE 3:**
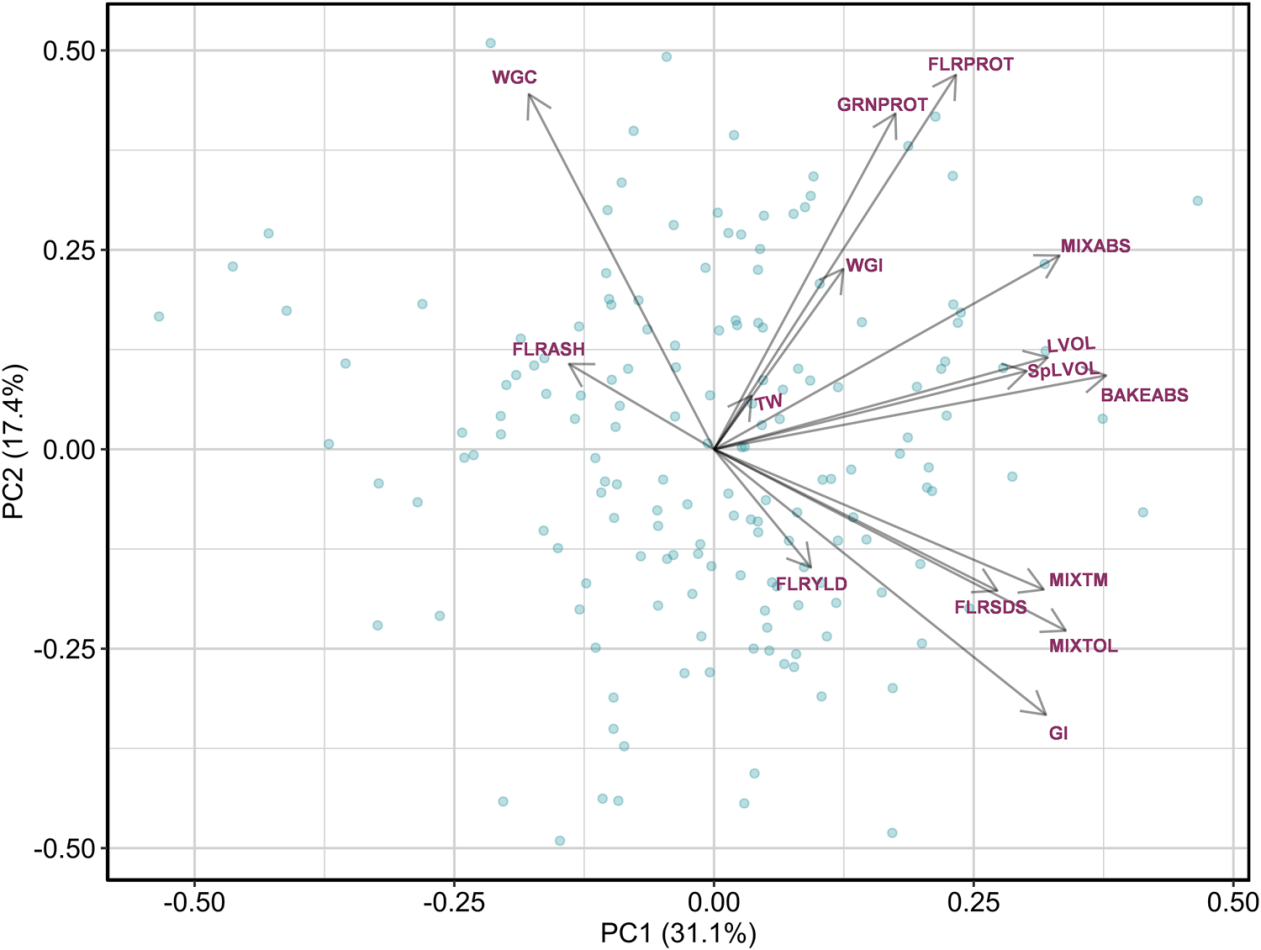
Principal component analysis for the studied end‐use quality traits based on the phenotypic data. BAKEABS, bake absorption; FLRASH, flour ash content; FLRPROT, flour protein content; FLRSDS, flour sedimentation weight value; FLRYLD, flour yield; GI, gluten index; GRPROT, grain protein content; LVOL, pup loaf volume; MIXABS, mixograph mixing absorption; MIXTM, mixograph mix time; MIXTOL, mixograph mix tolerance score; SpLVOL, specific loaf volume by weight; TW, test weight; WGC, wet gluten content; WGI, wet gluten index.

PCA using all the trait data also showed similar groups of correlated traits (Figure 3). The first and second components from PCA explained 31.1% and 17.4% of the total phenotypic variance. PCA showed a strong association between GRPROT and FLRPROT. The baking traits were found to be positively associated with MIXABS, whereas another group included MIXTM, MIXTOL, GI, and FLRSDS. Further, we estimated the genetic correlations among different traits using the Bayesian Multivariate Gaussian model (Figure S3). Interestingly, FLRSDS exhibited significant genetic correlations with different quality parameters (*p* ≤ 0.001), including GI (0.71), MIXTM (0.49), MIXTOL (0.56), BAKEABS (0.47), and LVOL (0.44). Apart from FLRSDS, FLRPROT showed significant genetic correlations with MIXABS (0.78), BAKEABS (0.62), and LVOL (0.43) (Figure S3).

### 3.2 Genotypic analyses

The breeding lines were genotyped using the GBS markers. A total of 8725 high‐quality SNPs markers were obtained covering all 21 wheat chromosomes. PCA was performed using 8725 SNPs to investigate relationships among studied lines (Figure S4). The first and second components explained 6.6% and 5.5% of the total variance, respectively. Overall, the absence of strong clustering based on PCA suggested close relationships between the lines used in this study indicating the suitability of this set of wheat breeding lines for evaluating GP models.

### 3.3 Predictive ability of single trait models

**FIGURE 4:**
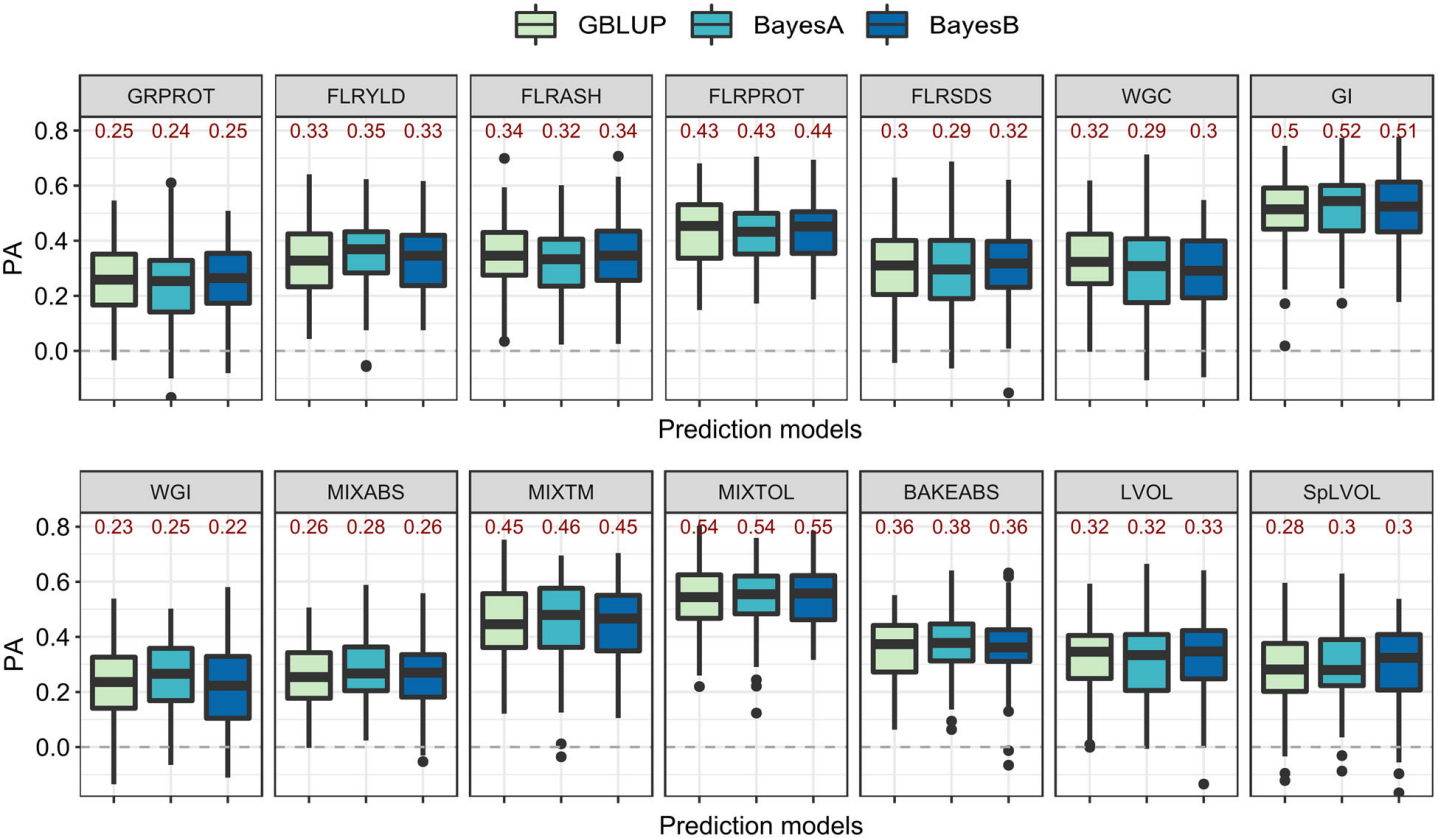
Prediction ability of different single‐trait genomic prediction (GP) models for 14 end‐use quality traits in cross‐validation. BAKEABS, bake absorption; FLRASH, flour ash content; FLRPROT, flour protein content; FLRSDS, flour sedimentation weight value; FLRYLD, flour yield; GI, gluten index; GRPROT, grain protein content; LVOL, pup loaf volume; MIXABS, mixograph mixing absorption; MIXTM, mixograph mix time; MIXTOL, mixograph mix tolerance score; PA, prediction accurary; SpLVOL, specific loaf volume by weight; WGC, wet gluten content.

We used STGP to assess the PA of end‐use quality traits using the CV1 scheme of cross‐validation (Figure S2). Three different single‐trait models (GBLUP, BA, and BB) were compared to identify the baseline for the evaluation of different MT models. Overall, we did not observe any difference in the performance of these three ST models as all yielded comparable PA for different traits (Figure 4; Table S3). Thus, the univariate GBLUP model (ST‐GBLUP hereafter) was selected as a baseline to compare with the MT models. PAs using ST‐GBLUP varied from 0.23 to 0.54 for different end‐use quality traits, with the lowest PA for MIXABS (0.26), and the highest PA for MIXTOL (0.54) (Figure 4).

### 3.4 MT models to predict mixograph and Glutomatic traits

**FIGURE 5:**
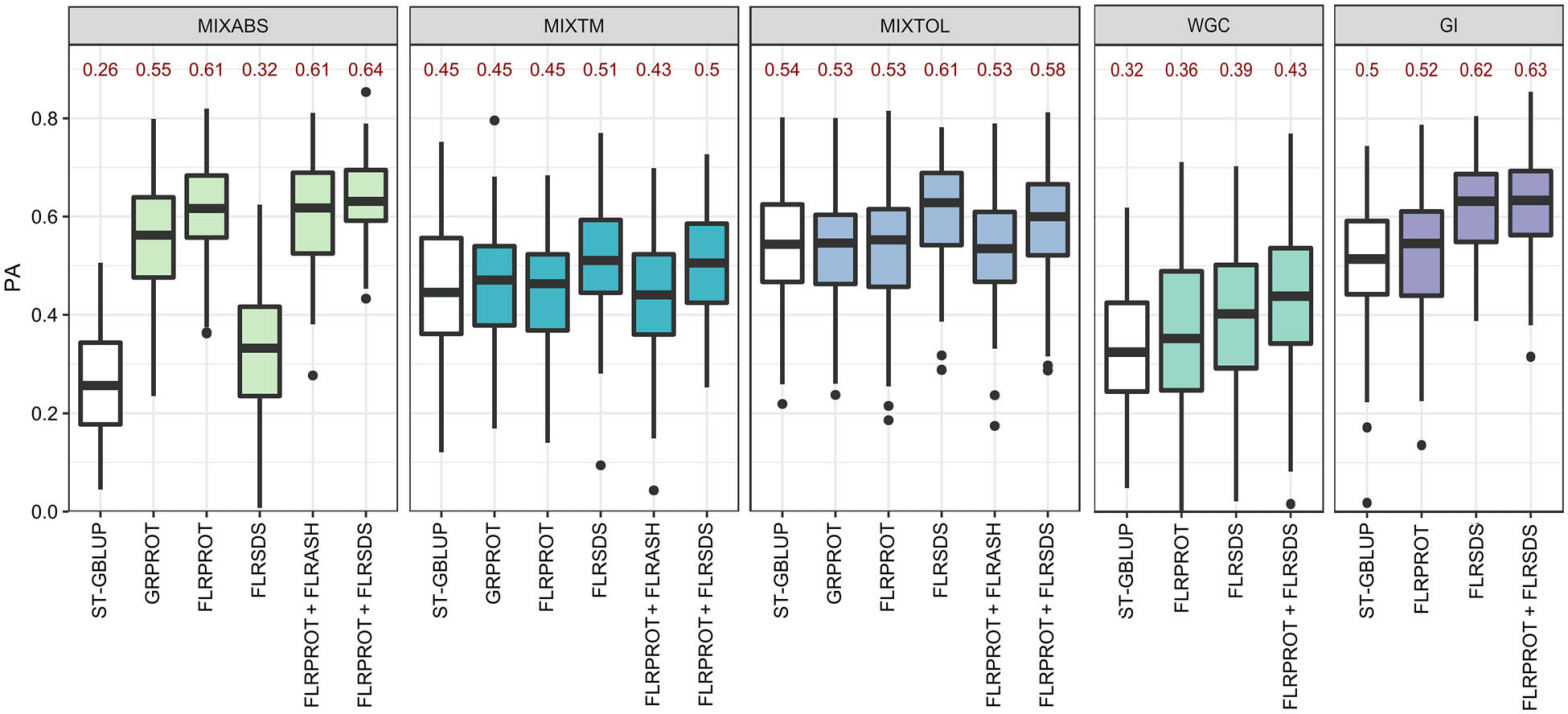
Prediction ability of the multi‐trait genomic prediction (MTGP) model for various mixograph and Glutomatic traits using different combinations of secondary traits. The ST‐GBLUP refers to the baseline single‐trait GP model for the respective trait. FLRASH, flour ash content; FLRPROT, flour protein content; FLRSDS, flour sedimentation weight value; GBLUP, genomic best linear unbiased predictor; GI, gluten index; GRPROT, grain protein content; MIXABS, mixograph mixing absorption; MIXTM, mixograph mix time; MIXTOL, mixograph mix tolerance score; PA, prediction accuracy; WGC, wet gluten content.

We evaluated the ability of MTGP models to predict a primary trait of interest by incorporating less resource‐intensive secondary traits as covariates in the MTGP model and to determine the most effective combinations. The MT model was used to predict mixograph traits, Glutomatic traits, and baking traits, which otherwise are time and resource intensive to phenotype. We evaluated the PA for three important mixograph traits (MIXABS, MIXTM, and MIXTOL) using the MT model with secondary traits from rapid assays such as FLRPROT. A considerable improvement in PA for MIXABS was observed when the MT model was used with different combinations of secondary traits (Figure 5; Table S4). The PA for MIXABS was 0.61 when FLRPROT was used as a secondary trait, and 0.64 when FLRPROT and FLRSDS were used together, which was higher than the PA for MIXABS using ST‐GBLUP (0.26). For MIXTM, the MT model using FLRSDS as covariate yielded higher PA (0.51) compared to the ST‐GBLUP model (0.45). Similarly, the inclusion of FLRSDS in MT model yielded the highest PA (0.61) for MIXTOL. Overall, the inclusion of FLRPROT and FLRSDS as the secondary traits in the MT model was effective to predict different Mixograph traits (Figure 5; Table S4).

Further, we evaluated different combinations of secondary traits to identify the best set of covariates to predict Glutomatic traits. We used three combinations (FLRPROT, FLRSDS, or FLRPROT + FLRSDS) of secondary traits to predict WGC and GI using the MT model (Figure 5). The PA for WGC was the highest (0.43) when FLRPROT + FLRSDS were used as the covariates in the MT model, which was a substantial improvement over the ST‐GBLUP model (0.32). For GI, the inclusion of FLRPROT + FLRSDS in the MT model yielded a PA of 0.63 compared to 0.50 for the ST‐GBLUP model (Table S5).

### 3.5 MT models to predict baking traits

**FIGURE 6:**
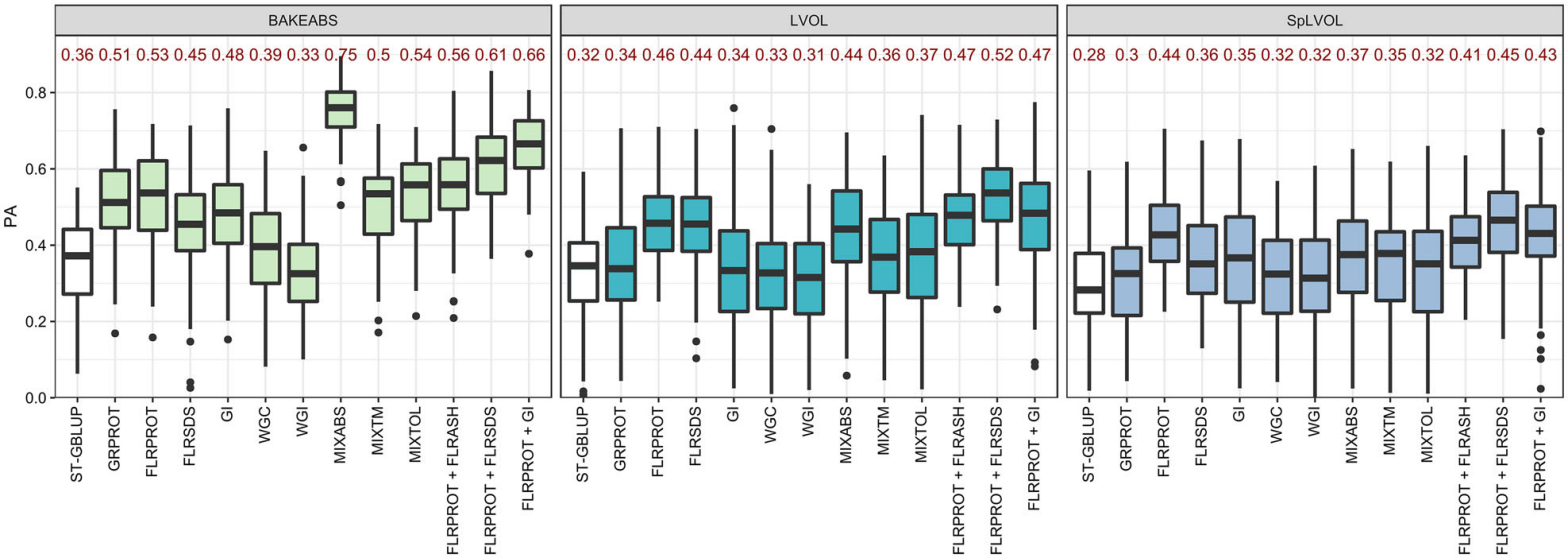
Prediction ability of the multitrait (MT) genomic prediction model for various baking traits using different combinations of secondary traits. The ST‐GBLUP refers to the baseline single‐trait genomic prediction (GP) model for the respective trait. BAKEABS, bake absorption; FLRASH, flour ash content; FLRPROT, flour protein content; FLRSDS, flour sedimentation weight value; GBLUP, genomic best linear unbiased predictor; GI, gluten index; GRPROT, grain protein content; LVOL, pup loaf volume; MIXABS, mixograph mixing absorption; MIXTM, mixograph mix time; MIXTOL, mixograph mix tolerance score; SpLVOL, specific loaf volume by weight; WGC, wet gluten content; WGI, wet gluten index.

BAKEABS and LVOL are important traits used by breeders to assess the end‐use quality of yeast‐leavened bread. Intending to predict the baking traits (BAKEABS, LVOL, and SpLVOL), we evaluated various combinations of secondary traits in the MT model (Table S6). These combinations were designed by selecting traits from different prebaking assays performed at various scales. The ST‐GBLUP showed a PA of 0.36 for BAKEABS (Figure 4), whereas a high PA (0.75) was observed when the MIXABS was included in the MT model (Figure 6, Table S6). Intriguingly, the incorporation of two easy‐to‐score traits, FLRPROT and FLRSDS, showed a high PA of 0.61. However, the addition of Glutomatic traits did not show any substantial improvement (0.66) in PA for BAKEABS (Figure 6, Table S6).

The PA for LVOL ranged from 0.31 to 0.52 using different combinations of covariates in the MTGP model (Figure 6, Table S6). The highest PA (0.52) for LVOL was obtained when both FLRPROT and FLRSDS were included in the MT model, which was considerably higher than the ST‐GBLUP model (0.32) and also higher than the inclusion of only FLRPROT or FLRSDS in the MT model (Table S6). Similar to BAKEABS, including Glutomatic traits in the MT model did not improve the PA for LVOL (Figure 6). For SpLVOL, the PA ranged from 0.30 to 0.45 (Figure 6) using the MT model with the highest PA from the inclusion of both FLRPROT and FLRSDS.

## 4 DISCUSSION

In HWW breeding, end‐use quality and processing traits are important factors in varietal development and determining acceptance by the industry. However, intensive selection for these traits is restricted to later generations in a breeding program because of expensive and time‐consuming phenotyping requirements. Moreover, a short turnaround time in winter wheat breeding program creates another challenge in performing quality evaluations. For instance, winter wheat breeders particularly from the northern Great Plains get only a month between the harvest and planting to make selections, seed preparation, and turn around the breeding cycle, which eliminates the possibility to conduct comprehensive quality analysis on the lines that are to be advanced. This limits the selection decisions of quality traits to be made based on some easily measured traits or preliminary data obtained from prior year nurseries. GS may provide an alternative strategy to estimate GEBVs for these traits to cull inferior lines in earlier generations (Battenfield et al., 2016; Belamkar et al., 2018; Gill et al., 2021; Ward et al., 2019). However, conventional univariate GP models have shown a weak PA for complex end‐use quality traits owing to their complex genetic architecture and low heritabilities (Battenfield et al., 2016; Sandhu, Aoun, et al., 2021; Zhang‐Biehn et al., 2021). In recent years, MTGP models have been proposed to improve the PA of complex traits when phenotypic data for secondary traits are available (Bhatta et al., 2020; Gaire et al., 2022; Jia & Jannink, 2012; Lado et al., 2018; Zhang et al., 2022). In this study, we used MTGP models and evaluated their PA for various end‐use quality traits measured at different stages of the breeding program. Rather than limiting to just model comparison, we evaluated several combinations of traits that can be used as covariates in the MTGP models to predict complex traits like BAKEABS and LVOL. Furthermore, we restricted the choice of secondary traits for MT models to the traits that are easy to score, inexpensive, and can be assessed on a large number of lines in early generations (Figure 1). This is the first study in HWW to evaluate MTGP models with these combinations of secondary traits for their use in forward prediction.

We observed significant variation in the phenotypic distribution of all 14 end‐use quality traits. Broad‐sense heritability estimates for most of the traits were moderate, with a few traits exhibiting high heritability (Table 1), which corroborated most findings from previous studies (Battenfield et al., 2016; Michel et al., 2018; Sandhu, Aoun, et al., 2021; Sandhu et al., 2021, 2022; Zhang‐Biehn et al., 2021). Traits, including MIXTM, MIXTOL, GI, and FLRSDS, had moderate‐to‐high heritability, suggesting that most variation associated with these traits can be attributed to genetic factors. As expected, all three baking traits showed low‐to‐moderate heritability (Table 1). This suggests the possibility of leveraging highly heritable traits in the multitrait models to predict traits like BAKEABS and LVOL. Further, we observed significant phenotypic and genetic correlations among a few pairs of traits (Figures 2 and 3). However, varying degrees of correlations among different quality tests suggested that no single test can completely substitute for actual testing of end‐use quality traits (Battenfield et al., 2016; Souza et al., 2002). Intriguingly, a rapid flour sedimentation test (FLRSDS) was found to be significantly correlated with various Glutomatic, mixograph, and baking traits. FLRSDS also exhibited the highest positive correlation with LVOL (Figure S3) in corroboration with Seabourn et al. (2012). In contrast to Seabourn et al. (2012), the correlation between FLRSDS and FLRPROT was not significant in our study, which might be due to the fact that hybrid SDS–SRC assay was performed in year 3 using residual grain stored in the lab. As rapid tests for FLRSDS can be performed on a large scale along with NIR for FLRPROT, the genetic correlation of these traits with LVOL can be exploited in MTGP models to select quality traits in early generations.

Three univariate GP models were evaluated for predicting various end‐use quality traits and selecting the best model for comparison with MT models. We did not observe significant differences in the performance among the univariate models GBLUP, BA, and BB. Previous studies have also reported similar outcomes while predicting complex traits (Sandhu, Aoun, et al., 2021; Sandhu, Patil, et al., 2021; Zhang et al., 2022). Further, most end‐use quality traits had low‐to‐moderate PA using ST models (Figure 4). Interestingly, the mixograph traits, MIXTM and MIXTOL, showed better PA using the ST model when compared with other traits likely because of their high broad‐sense heritabilities.

As discussed earlier, breeding programs rely on various types of quality assays during different stages of variety development to select lines with desirable end‐use quality (Figure 1). Quality tests, including mixograph, Glutomatic analysis, and baking, are resource‐ and cost‐intensive and can only be conducted in later generations of cultivar development due to limited grain quantity available in early generations (Figure 1). We used the MTGP model to assess the PA of various traits from these analyses using different combinations of secondary traits (FLRPROT and FLRSDS) that can be analyzed rapidly. Overall, the MT model outperformed the ST‐GBLUP model for all the traits evaluated in this study (Figure 6). However, the extent of improvement using the MT model varied for different sets of traits. In corroboration with previous studies (Arojju et al., 2020; Gill et al., 2021; Rutkoski et al., 2016; Sun et al., 2017; Zhang et al., 2022), the improvement in PA using the MT model relied on various factors, including the heritability of primary and secondary traits, different combinations of secondary traits, and genetic correlation between primary and secondary traits.

The ST‐GBLUP model yielded a PA of 0.26, 0.45, and 0.54 for MIXABS, MIXTM, and MIXTOL, respectively (Figure 4). We used the MT model using flour characteristics from NIR analysis and FLRSDS from the hybrid SDS–SRC test as secondary traits to predict MIXABS, MIXTM, and MIXTOL. For MIXABS, the MT model yielded a PA of 0.64 when FLRPROT and FLRSDS were used as the covariates, resulting in more than a twofold increase in PA over the ST‐GBLUP model. We also observed an improvement in PA using the MT model for MIXTIM and MIXTOL, and the highest PA was observed while using FLRPROT and FLRSDS as secondary traits (Figure 5). The improvement in PA for MIXTOL was less than for MIXABS and MIXTIM when the MT model was used. This relates to the high heritability observed for MIXTOL, suggesting that the MT models are more useful for traits exhibiting low heritability and thus lower PA when using the ST models (Gill et al., 2021; Jia & Jannink, 2012; Rutkoski et al., 2016; Ward et al., 2019). Similar to mixograph, the use of MTGP outperformed the ST‐GBLUP model in predicting GI and WGC with both FLRPROT and FLRSDS being the most effective combination of covariates.

The primary end‐use product made from HWW is yeast‐leavened bread. BAKEABS and LVOL are important traits used by breeders to assess end‐use quality potential. However, the evaluation of these traits is restricted to later‐generation elite materials only. Thus, GS can be a promising approach to inform selection based on these traits in early generations. The MT model outperformed ST‐GBLUP for predicting all baking traits (Figure 6). For BAKEABS, we observed a PA of 0.75 when MIXABS was included in the MT model. Nevertheless, the inclusion of both FLRPROT and FLRSDS yielded a PA of 0.61, which was higher than the ST‐GBLUP model (0.36). The mixograph analysis has been a regular practice for assessing dough properties of the mid‐to‐late‐generation samples of the HWW breeding programs. The mixograph procedure requires only a small quantity of flour (10 g) and various mixograph parameters are used to infer the mixing properties of flour and estimate the breadmaking water absorption and LVOL without actual baking. However, the recent discontinuation of the mixograph instrument and unavailability of alternatives will limit the breeder's capacity to get this information in earlier stages. Our results suggest that MTGP can provide an opportunity to directly predict BAKEABS and other baking traits using information from simple assays like FLRPROT and FLRSDS and potentially offset the gap created due to the discontinuation of the mixograph. Further, the inclusion of both FLRPROT and FLRSDS resulted in higher PA for LVOL and SpLVOL compared to other combinations of secondary traits (Figure 6). Results from this study suggest that the MTGP models can effectively predict various end‐use quality traits, including baking traits. Moreover, a combination of secondary traits (FLRPROT and FLRSDS) resulted in substantial improvement in PA for baking traits. A previous HWW study also reported that the inclusion of GRPROT or FLRPROT improved the PA for mixograph and baking traits, whereas the inclusion of other traits as covariates was not useful (Zhang‐Biehn et al., 2021).

It is also noteworthy that the MTGP will be useful if it performs better than the routine indirect selection of primary trait(s) using highly correlated secondary traits. Except for Mixograph traits, we observed that the predictive abilities using MT models were higher than the phenotypic correlations between primary and secondary trait(s), especially for important baking traits (LVOL and BAKEABS). Additionally, it is expected that further optimization of training populations and increased data points will further improve the PA of MT models. Overall, our results suggest that the MTGP for end‐use quality traits such as LVOL can be a part of the GP pipeline of the HWW breeding program along with other agronomic traits.

## 5 CONCLUSION

In conclusion, wheat breeding programs can routinely perform rapid assays such as NIR‐based characterization of flour or sedimentation tests in an earlier generation of a breeding program. These assays are cost‐effective and do not require intensive resources. For instance, the hybrid SDS–SRC for FLRSDS can be performed using 1 g of whole wheat flour, and 25–30 samples can be evaluated per hour (Seabourn et al., 2012). Similarly, flour characteristics such as FLRPROT can be quickly assayed using NIR‐based spectroscopy, and some newer NIRs also provide an estimation of absorption and GI, which can be further evaluated. Further, the availability of low‐cost genotyping platforms has made it possible for breeding programs to sequence a large number of lines in earlier generations. Thus, the availability of these resources can help to implement multi‐trait genomic selection (MTGS) for predicting traits like LVOL that are difficult to phenotype and exhibit low heritability. The MTGS models can be employed by combining FLRPROT and FLRSDS evaluated from earlier generations with a complete quality profile (including baking) from the advanced generations to predict baking traits in earlier generations (PYT or EOT). This will not only save considerable time and resources but will provide an opportunity for breeders to eliminate inferior material in earlier generations. Therefore, MTGS holds great promise in improving the selection efficiency of processing and end‐use quality traits in HWW.

## AUTHOR CONTRIBUTIONS

**Harsimardeep S. Gill**: Conceptualization; Data curation; Formal analysis; Investigation; Methodology; Software; Validation; Visualization; Writing‐original draft. **Navreet Brar**: Investigation; Writing‐review & editing. **Jyotirmoy Halder**: Investigation; Writing‐review & editing. **Cody Hall**: Investigation. **Bradford W. Seabourn**: Investigation; Writing‐review & editing. **Yuanhong R. Chen**: Investigation; Writing‐review & editing. **Paul St. Amand**: Investigation; Software; Writing‐review & editing. **Amy Bernardo**: Investigation; Writing‐review & editing. **Guihua Bai**: Formal analysis; Investigation; Writing‐review & editing. **Karl Glover**: Writing‐review & editing. **Brent Turnipseed**: Writing‐review & editing. **Sunish K. Sehgal**: Conceptualization; Data curation; Funding acquisition; Methodology; Project administration; Resources; Supervision; Validation; Writing‐original draft; Writing‐review & editing.

## CONFLICT OF INTEREST STATEMENT

The authors declare that the research was conducted in the absence of any commercial or financial relationships that could be construed as a potential conflict of interest.

## SUPPLEMENTARY MATERIAL

The Supplementary information cited in the manuscript can be found in an attached PDF document.

## Supporting information

Supporting Information

## Data Availability

The datasets generated during this study can be found in this article and its supplementary information files.
