# Supplementary material for: Multi‐trait genomic selection improves the prediction accuracy of end‐use quality traits in hard winter wheat
Source: Plant Genome. 2023 May 17;16(4):e20331. doi: 10.1002/tpg2.20331 (PMC12806972; doi:10.1002/tpg2.20331)
Supplement: Supplementary file 1 [file TPG2-16-e20331-s001.pdf]

Supplementary Information

Multi-trait genomic selection improves the prediction accuracy of end-use quality traits in hard winter wheat

Harsimardeep S. Gill[1], Navreet Brar[1], Jyotirmoy Halder[1], Cody Hall[1], Bradford W Seabourn[2], Yuanhong R Chen[2], Paul St. Amand[3], Amy Bernardo[3], Guihua Bai[3], Karl Glover[1], Brent Turnipseed[1], Sunish K. Sehgal[1]*

Supplementary Figures

Supplementary Figure S1. Schematic representation of different combinations of secondary traits used in the MT model to predict primary traits of interest. The diagram illustrates a scenario to predict LVOL (primary trait) using the MT model. Various combinations of secondary traits were selected based on different types of pre-baking assays, such as grain/flour characteristics or a flour sedimentation test, performed at various levels in a breeding program. The training set had phenotype data for LVOL while the validation set was not phenotyped for this trait. Contrarily, phenotype data or secondary trait(s) were available for both testing and validation sets.

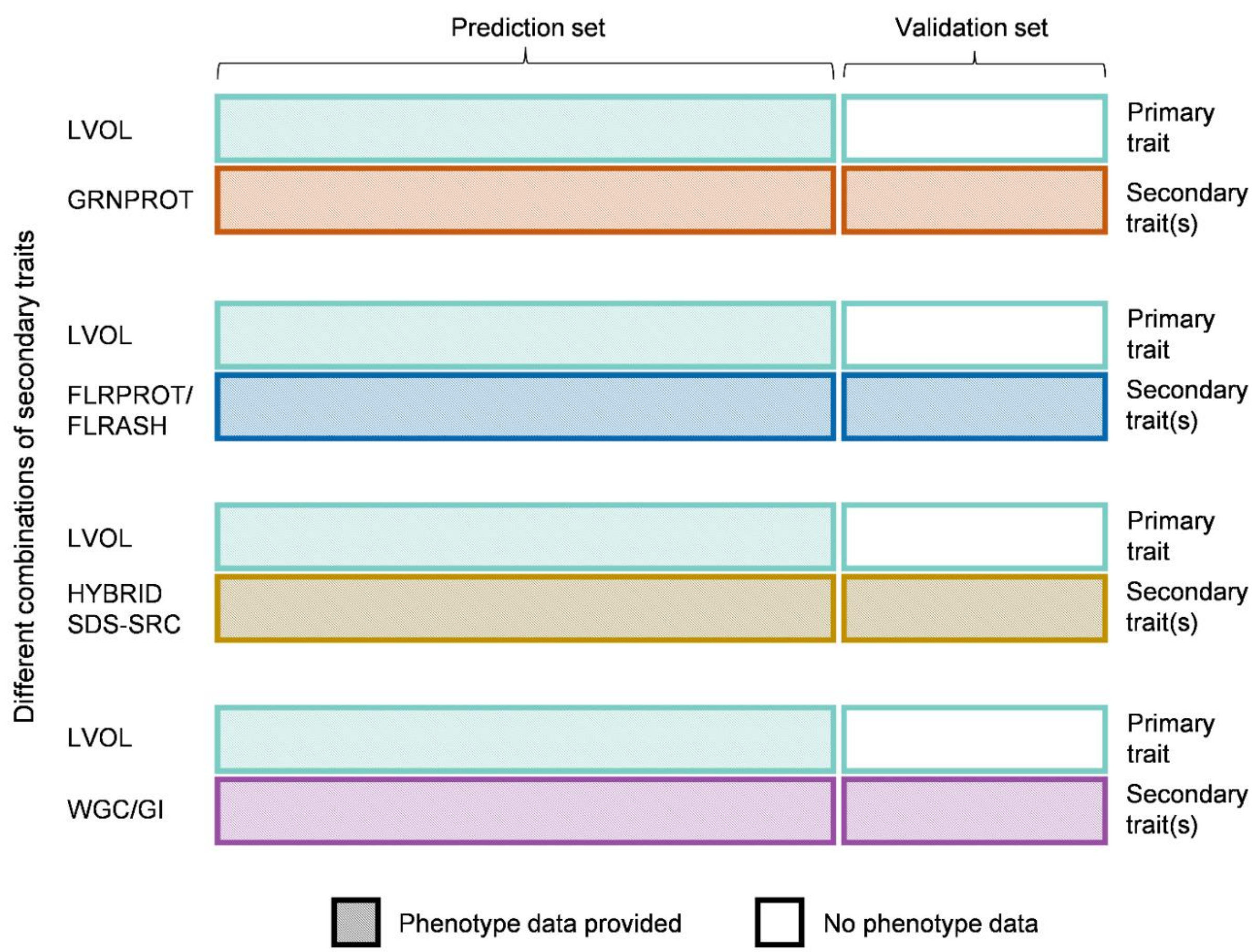

Supplementary Figure S2. Illustration of the different cross-validation (CV) schemes used in this study. The Single trait (ST) Model was evaluated using a CV1 scheme where four sets were used to train the model and the remaining set was used as a testing/validation set. The training set had phenotypic data and genotypic data while the testing data had only genotypic data. The MT model was evaluated using CV2 scheme. In CV2, the training set had phenotyped for the primary trait (trait to be predicted) while the validation set was not phenotyped for this trait. Contrarily, phenotype data or secondary trait(s) was available for both testing and validation sets.

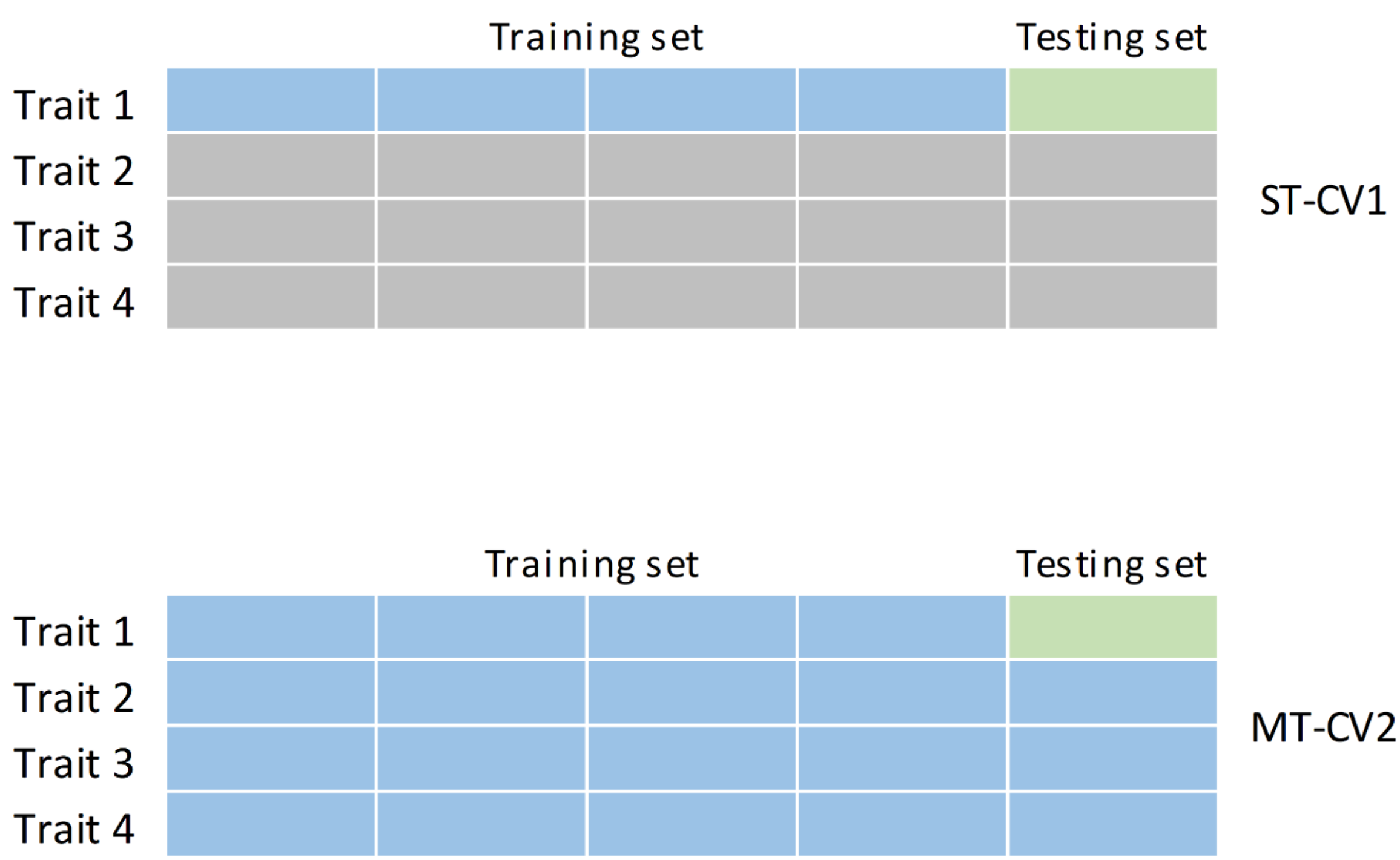

Supplementary Figure S3. The genetic correlation among various end-use quality traits. GRPROT, grain protein content; FLRYLD, flour yield; FLRASH, flour ash content; FLRPROT, flour protein content; FLRSDS, flour sedimentation weight; WGC, wet gluten content; GI, gluten index; MIXABS, Mixograph mixing absorption, MXTIM, Mixograph mix time; MIXTOL, Mixograph mix tolerance score; BAKEABS, bake absorption; LVOL, pup loaf volume.

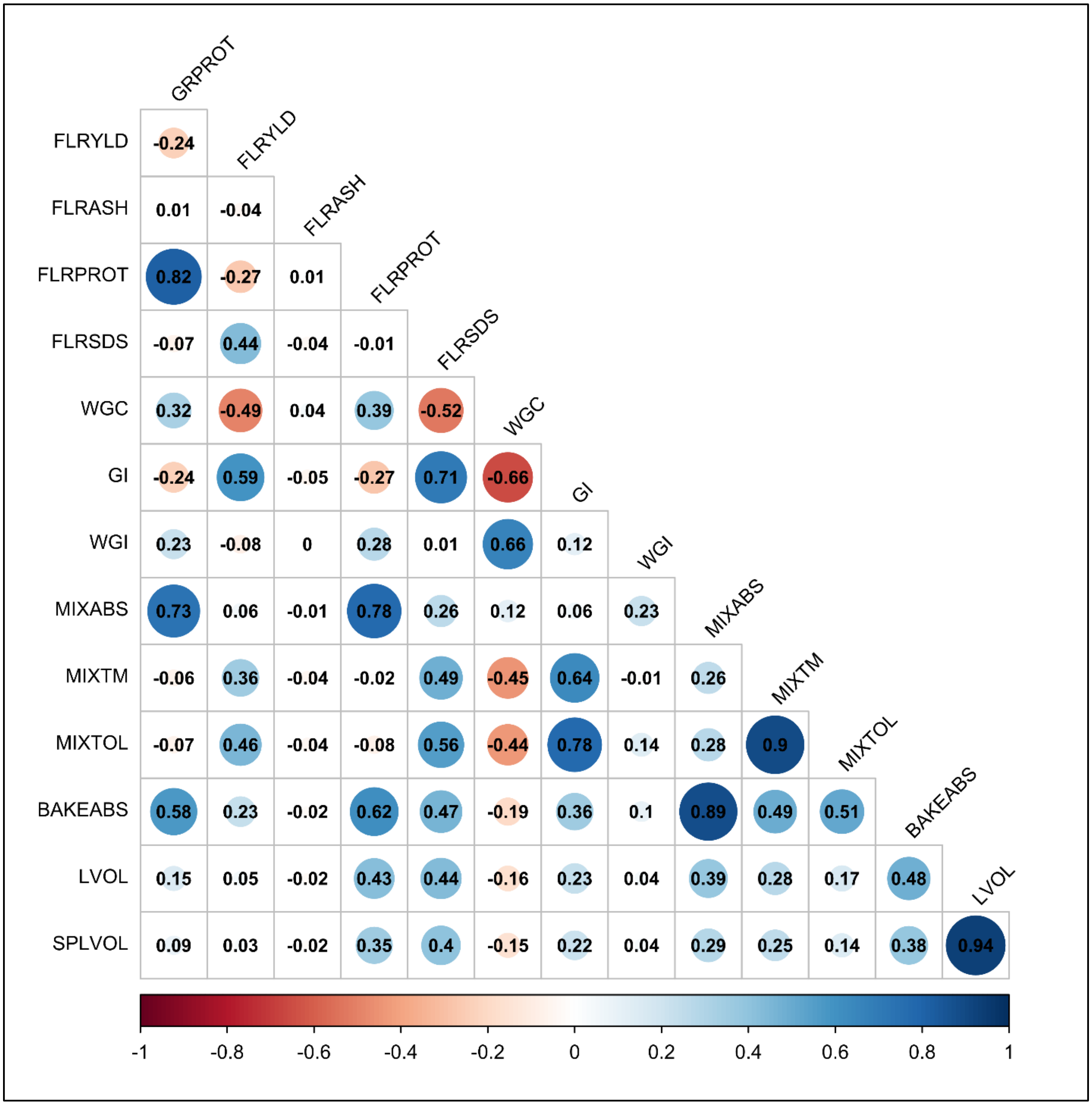

Supplementary Figure S4. Principal component analysis for studied lines based on 8,725 single nucleotide polymorphism (SNP) markers.

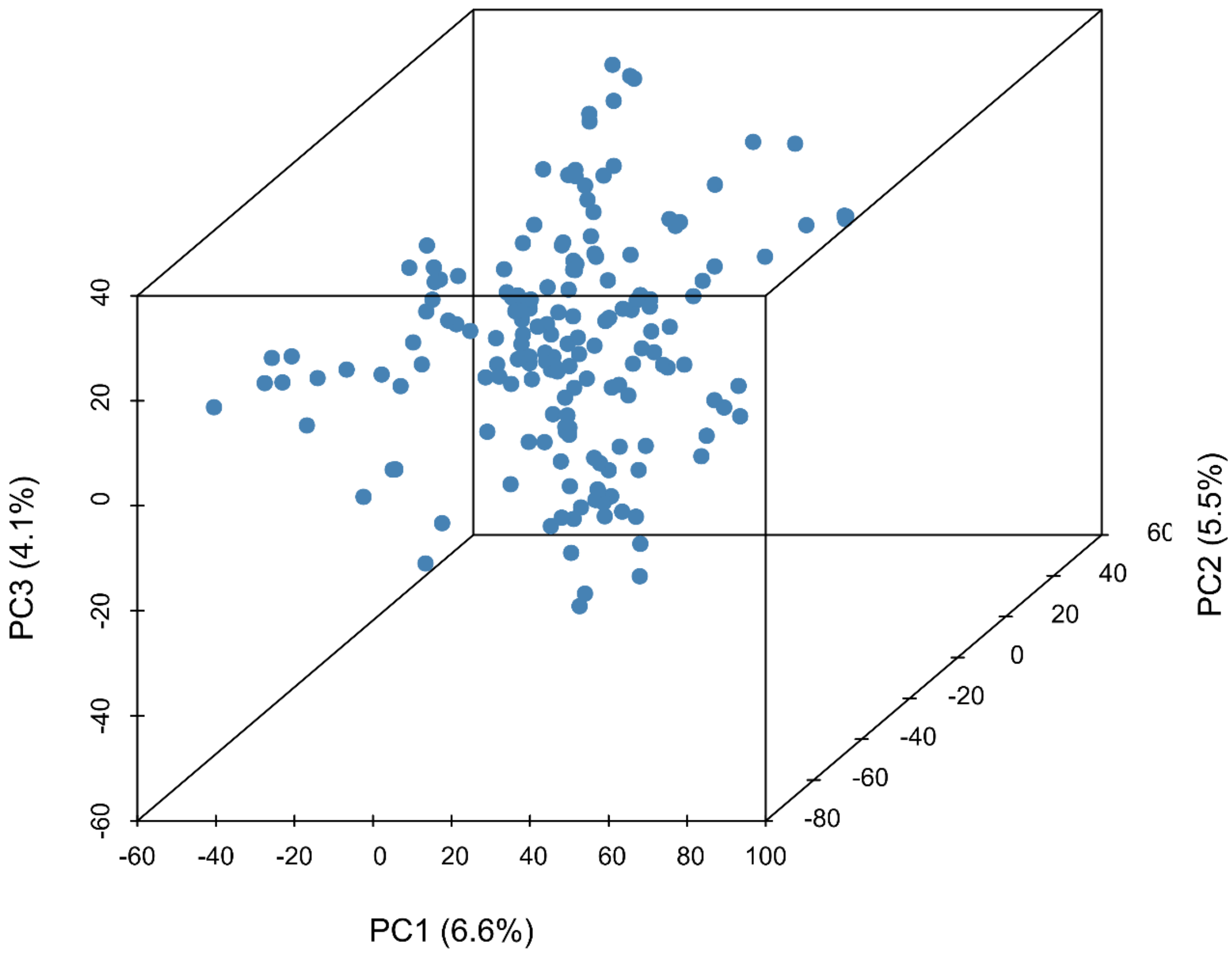

Supplementary Tables

Supplementary Table S1. Description of trial years for the breeding lines used in the current study.

Year of evaluation	Nurseries[a]	Number of Entries	Number of locations	Entries overlapping with the previous year
2015	EYT, CPT	35	1	-
2016	EYT, CPT	39	2	18
2017	EYT, CPT	43	2	19
2018	EYT, CPT	46	2	42
2019	EYT, CPT	47	2	14
2020	EYT, CPT	44	2	15
2021	EYT, CPT	46	2	10
	Total	300	13	-

[a]Nurseries: Elite Yield Trial, EYT; Crop Performance Trial, CPT.

Supplementary Table S2. Phenotypic correlations among all 14 end-use quality traits. GRPROT, grain protein content; FLRYLD, flour yield; FLRASH, flour ash content; FLRPROT, flour protein content; FLRSDS, flour sedimentation weight value; WGC, wet gluten content; GI, gluten index; WGI, wet gluten index; MIXABS, Mixograph mixing absorption, MXTIM, Mixograph mix time; MIXTOL, Mixograph mix tolerance score; BAKEABS, bake absorption; LVOL, pup loaf volume; SpLVOL, loaf volume by weight.

	GRPROT	FLRYLD	FLRASH	FLRPROT	FLRSDS	WGC	GI	WGI	MIXABS	MIXTM	MIXTOL	BAKEABS	LVOL	SpLVOL
GRPROT	1.00	-0.13	0.00	0.75	-0.04	0.18	-0.09	0.17	0.57	0.08	0.06	0.48	0.19	0.15
FLRYLD	-0.13	1.00	-0.29	-0.10	0.15	-0.14	0.22	-0.01	0.10	0.11	0.14	0.12	0.06	0.03
FLRASH	0.00	-0.29	1.00	0.01	-0.15	0.15	-0.26	-0.07	-0.09	-0.21	-0.19	-0.08	-0.13	-0.14
FLRPROT	0.75	-0.10	0.01	1.00	0.02	0.27	-0.12	0.25	0.65	0.14	0.06	0.53	0.44	0.40
FLRSDS	-0.04	0.15	-0.15	0.02	1.00	-0.38	0.56	0.12	0.25	0.31	0.41	0.34	0.34	0.33
WGC	0.18	-0.14	0.15	0.27	-0.38	1.00	-0.64	0.51	-0.03	-0.36	-0.41	-0.24	-0.15	-0.15
GI	-0.09	0.22	-0.26	-0.12	0.56	-0.64	1.00	0.29	0.22	0.54	0.72	0.39	0.29	0.28
WGI	0.17	-0.01	-0.07	0.25	0.12	0.51	0.29	1.00	0.20	0.07	0.22	0.12	0.16	0.16
MIXABS	0.57	0.10	-0.09	0.65	0.25	-0.03	0.22	0.20	1.00	0.32	0.36	0.76	0.40	0.31
MIXTM	0.08	0.11	-0.21	0.14	0.31	-0.36	0.54	0.07	0.32	1.00	0.77	0.47	0.31	0.29
MIXTOL	0.06	0.14	-0.19	0.06	0.41	-0.41	0.72	0.22	0.36	0.77	1.00	0.52	0.27	0.25
BAKEABS	0.48	0.12	-0.08	0.53	0.34	-0.24	0.39	0.12	0.76	0.47	0.52	1.00	0.46	0.36
LVOL	0.19	0.06	-0.13	0.44	0.34	-0.15	0.29	0.16	0.40	0.31	0.27	0.46	1.00	0.98
SpLVOL	0.15	0.03	-0.14	0.40	0.33	-0.15	0.28	0.16	0.31	0.29	0.25	0.36	0.98	1.00

Supplementary Table S3. The mean prediction ability along with standard error (SE) for 14 end-use quality traits using different single-trait genomic prediction models. GRPROT, grain protein content; FLRYLD, flour yield; FLRASH, flour ash content; FLRPROT, flour protein content; FLRSDS, flour sedimentation weight value; WGC, wet gluten content; GI, gluten index; WGI, wet gluten index; MIXABS, Mixograph mixing absorption (%), MXTIM, Mixograph mix time (min); MIXTOL, Mixograph mix tolerance score; BAKEABS, bake absorption; LVOL, pup loaf volume; SpLVOL, loaf volume by weight.

Trait	GBLUP		BayesA		BayesB	
	PA	SE	PA	SE	PA	SE
GRPROT	0.25	0.014	0.24	0.015	0.25	0.013
FLRYLD	0.33	0.014	0.35	0.013	0.33	0.012
FLRASH	0.34	0.012	0.32	0.013	0.34	0.013
FLRPROT	0.43	0.013	0.43	0.011	0.44	0.011
FLRSDS	0.30	0.014	0.29	0.015	0.32	0.013
WGC	0.32	0.014	0.29	0.016	0.30	0.014
GI	0.50	0.014	0.52	0.012	0.51	0.014
WGI	0.23	0.014	0.25	0.013	0.22	0.016
MIXABS	0.26	0.012	0.28	0.012	0.26	0.012
MIXTM	0.45	0.014	0.46	0.015	0.45	0.014
MIXTOL	0.54	0.012	0.54	0.012	0.55	0.011
BAKEABS	0.36	0.011	0.38	0.012	0.36	0.012
LVOL	0.32	0.014	0.32	0.014	0.33	0.014
SpLVOL	0.28	0.014	0.30	0.013	0.30	0.014

Supplementary Table S4. The mean prediction ability along with standard error (SE) for Mixograph traits using MTGP model. GRPROT, grain protein content; flour ash content; FLRPROT, flour protein content; FLRSDS, flour sedimentation weight value; MIXABS, Mixograph mixing absorption, MXTIM, Mixograph mix time; MIXTOL, Mixograph mix tolerance score.

Trait	Secondary trait(s)	PA	SE
MIXABS	GRPROT	0.55	0.012
MIXABS	FLRPROT	0.61	0.010
MIXABS	FLRSDS	0.32	0.014
MIXABS	FLRPROT + FLRASH	0.61	0.011
MIXABS	FLRPROT + FLRSDS	0.64	0.008
MIXTM	GRPROT	0.45	0.013
MIXTM	FLRPROT	0.45	0.012
MIXTM	FLRSDS	0.51	0.011
MIXTM	FLRPROT + FLRASH	0.43	0.014
MIXTM	FLRPROT + FLRSDS	0.50	0.011
MIXTOL	GRPROT	0.53	0.011
MIXTOL	FLRPROT	0.53	0.013
MIXTOL	FLRSDS	0.61	0.011
MIXTOL	FLRPROT + FLRASH	0.53	0.011
MIXTOL	FLRPROT + FLRSDS	0.58	0.011

Supplementary Table S5. The mean prediction ability along with standard error (SE) for Glutomatic traits using MTGP model. FLRPROT, flour protein content; FLRSDS, flour sedimentation weight; WGC, wet gluten content; GI, gluten index.

Trait	Secondary trait(s)	PA	SE
WGC	FLRSDS	0.39	0.014
WGC	FLRPRO	0.36	0.016
WGC	FLRPRO + FLRSDS	0.43	0.015
GI	FLRSDS	0.62	0.010
GI	FLRPRO	0.52	0.013
GI	FLRPRO + FLRSDS	0.63	0.012

Supplementary Table S6. The mean prediction ability along with standard error (SE) for Glutomatic traits using MTGP model. GRPROT, grain protein content; FLRASH, flour ash content; FLRPROT, flour protein content; FLRSDS, flour sedimentation weight value; WGC, wet gluten content; GI, gluten index; WGI, wet gluten index; MIXABS, Mixograph mixing absorption, MXTIM, Mixograph mix time; MIXTOL, Mixograph mix tolerance score; BAKEABS, bake absorption; LVOL, pup loaf volume; SpLVOL, loaf volume by weight.

Trait	Secondary trait(s)	PA	SE
BAKEABS	GRPROT	0.51	0.012
BAKEABS	FLRPROT	0.53	0.012
BAKEABS	FLRSDS	0.45	0.013
BAKEABS	GI	0.48	0.012
BAKEABS	WGC	0.39	0.013
BAKEABS	WGI	0.33	0.011
BAKEABS	MIXABS	0.75	0.007
BAKEABS	MIXTM	0.50	0.012
BAKEABS	MIXTOL	0.54	0.010
BAKEABS	FLRPROT + FLRASH	0.56	0.011
BAKEABS	FLRPROT + FLRSDS	0.61	0.010
BAKEABS	FLRPROT + GI	0.66	0.009
LVOL	GRPROT	0.34	0.016
LVOL	FLRPROT	0.46	0.010
LVOL	FLRSDS	0.44	0.011
LVOL	GI	0.34	0.017
LVOL	WGC	0.33	0.015
LVOL	WGI	0.31	0.013
LVOL	MIXABS	0.44	0.013
LVOL	MIXTM	0.36	0.015
LVOL	MIXTOL	0.37	0.016
LVOL	FLRPROT + FLRASH	0.47	0.010
LVOL	FLRPROT + FLRSDS	0.52	0.010
LVOL	FLRPROT + GI	0.47	0.013
SpLVOL	GRPROT	0.30	0.014
SpLVOL	FLRPROT	0.44	0.011
SpLVOL	FLRSDS	0.36	0.012
SpLVOL	GI	0.35	0.016
SpLVOL	WGC	0.32	0.012
SpLVOL	WGI	0.32	0.014

SpLVOL	MIXABS	0.37	0.013
SpLVOL	MIXTM	0.35	0.013
SpLVOL	MIXTOL	0.32	0.017
SpLVOL	FLRPROT + FLRASH	0.41	0.010
SpLVOL	FLRPROT + FLRSDS	0.45	0.013
SpLVOL	FLRPROT + GI	0.43	0.012
